# Prevalence and risk predictors of onychomycosis in patients on hemodialysis: an observation, prospective, and unicenter study in Brazil

**DOI:** 10.3389/fmed.2023.1268324

**Published:** 2023-11-23

**Authors:** Jeanne Marie Queiroz Borges Bersano, Matheus Gobbo Cordeiro, Fernando Augusto Lima Marson

**Affiliations:** Laboratory of Molecular Biology and Genetics, Health Sciences Postgraduate Program, Universidade São Francisco, Bragança Paulista, São Paulo, Brazil

**Keywords:** dermatophyte filamentous fungi, gender, microbiology, non-dermatophyte filamentous fungi, obesity, onychomycosis, renal dialysis, yeast

## Abstract

**Background:**

Onychomycoses are fungal infections that can be seen in any component of the nail unit, including the matrix, bed, and plate, and are caused by dermatophyte fungi, non-dermatophyte fungi, and yeasts. This disease affects approximately 1 to 8% of the general population and occurs in approximately 19 to 51.9% of the patients on hemodialysis. The high incidence of onychomycosis in patients on hemodialysis is associated, mainly, with immunologic deficits and histological changes caused by uremia.

**Methods:**

Adult patients of the São Francisco University Hospital Hemodialysis Center were included. The following characteristics were evaluated: age, sex, body mass index, comorbidity, and household location. All patients were clinically evaluated and those with suspected onychomycosis had subungual debris of the affected nail plate collected for the direct mycological examination and fungal culture. The onychomycosis severity for those patients with a positive result in the fungal culture examination was evaluated using the Onychomycosis Severity Index system.

**Results:**

The study included 151 patients, and 70 out of the 151 patients (46.4%) showed nail alteration, and among them, 31 out of the 70 patients (44.3%) had the onychomycosis diagnosis confirmed by direct mycological examination. The pathogens observed in the patients were *Trichophyton rubrum* [8 out of 31 (25.8%)], *Trichophyton mentagrophytes* [7 out of 31 (22.6%)], S*cytalidium* spp. [6 out of 31 (19.4%)], *Candida* spp. [2 out of 31 (6.45%)], *Rhodotorula* spp. [1 out of 31 (3.2%)], *Candida albicans* [1/31 (3.2%)], *Penicillium marneffei* [1 out of 31 (3.22%)], and *T. rubrum* and *Rhodotorula* spp. [1 out of 31 (3.2%)]. Three participants presented negative results in the culture examination, and one did not allow the collection of material for the examination. The nail involvement severity score for the majority of them was severe [23 out of 27 (85.2%)], and only 1 out of the 27 (3.7%) and 3 out of the 27 (1.1%) patients presented moderate and mild scores, respectively. The distal subungual onychomycosis occurred in 12 out of 27 (44.4%) patients, a mixed pattern occurred in 14 out of 27 (51.9%) patients, and, white superficial occurred in only 1 out of 27 (3.7%) patients. In the bivariate analysis, a higher risk of onychomycosis was associated with the male sex [23/31 (74.2%) vs. 56/120 (46.7%); OR = 3.286 (95%CI = 1.362 to 7.928)] and obesity [8/31 (25.8%) vs. 12/120 (10.0%); OR = 3.130 (95%CI = 1.150 to 8.521)]. Patients with diabetes mellitus were more susceptible to onychomycosis attacks (*p*-value = 0.049; 16 out of 31 (51.6%) vs. 40 out of 120 (33.3%); however, OR was 2.133 (95%CI = 0.959 to 4.648). The patients with onychomycosis were older but without a significant difference between the groups (*p*-value = 0.073; 66 years old vs. 58 years old). The multivariable model using the logistic regression (backward model) confirmed our results and was able to predict (81.5%) the onychomycosis-positive diagnosis (chi-square = 27.73; *p*-value <0.001). The age [OR = 1.036; 95%CI = 1.004 to 1.069], male sex [OR = 5.746; 95%CI = 2.057 to 16.046], and presence of obesity [OR = 4.800; 95%CI = 1.435 to 16.055] were positive and significant in predicting the onychomycosis-positive diagnosis.

**Conclusion:**

In our study, onychomycosis in patients on hemodialysis was associated with a great variety of microorganisms, mainly *Trichophyton* species. The nail involvement severity score for the majority of patients was severe, and distal subungual onychomycosis and mixed pattern onychomycosis were the most prevalent clinical types. The main risk factors associated with onychomycosis were male sex, older age, and the presence of obesity.

## Highlights

1. Onychomycoses affect approximately 1 to 8% of the general population and occur in approximately 19 to 51.9% of the patients on hemodialysis.
2. A total of 70 out of 151 (46.4%) patients on hemodialysis showed nail alteration, and among them, 31 out of 70 (44.3%) had the onychomycosis diagnosis confirmed.
3. The pathogens observed in the patients on hemodialysis were *Trichophyton rubrum*, *Trichophyton mentagrophytes*, S*cytalidium* spp., *Candida* spp., *Rhodotorula* spp., *Candida albicans*, *Penicillium marneffei*, and *T. rubrum* and *Rhodotorula* spp.
4. The nail involvement severity score for the majority of them was severe.
5. Distal subungual onychomycosis and mixed pattern onychomycosis were the most prevalent clinical types.
6. A higher risk of onychomycosis in patients on hemodialysis was associated with male sex, older age, and obesity.

## 1 Introduction

Patients on hemodialysis experience several skin and nail alterations due to the systemic changes caused by the chronic renal disease and its etiologies (e.g., systemic arterial hypertension, diabetes mellitus, glomerulopathy, nephritis, dominant autosomal polycystic renal disease, and obstructive uropathy) (1–4). Among skin alterations, the main occurrences observed are hyperpigmentation, xerosis, pallor, itching, jaundice, plant hyperkeratosis, psoriasis, and viral, bacterial, or fungal infection complications (1–5). Onychomycosis, xerosis, and itching were found to occur simultaneously (1). Moreover, among nail alterations, the most frequent are onychomycosis, half and half nails, absent lunula, subungual hyperkeratosis, onycholysis, subungual hemorrhage, and *tinea pedis* (1–3, 6). Some alterations might occur simultaneously in the same patient such as onychomycosis and onycholysis and onycholysis and subungual hyperkeratosis (6).

Onychomycoses are nail fungal infections that can involve any component of the nail unit, including the matrix, bed, and plate, causing, for example, endonyx onychomycosis, proximal subungual onychomycosis, and total dystrophic onychomycosis (7). Onychomycoses are caused by dermatophyte filamentous fungi (e.g., *Trichophyton rubrum* and *Trichophyton mentagrophytes—*71%), non-dermatophyte fungi (e.g., *Scopulariopsis brevicaulis*, *Acremonium* spp., *Aspergillus* spp., *Fusarium* spp., and *Neoscytalidium* spp.—20.4%), or yeasts (*Candida* spp.—7.6%) (7–9). Dermatophyte filamentous fungi predominate in positive cultures in hemodialysis, followed by non-dermatophyte filamentous fungi, and yeasts (10–13). However, yeasts are more prevalent in older patients with diabetes mellitus and psoriasis (11).

Onychomycosis affects between 1 and 8% of the global population, with higher prevalence among those in hospital treatment (7, 9) and those on hemodialysis—19 to 51.9% (1–6, 10–13), representing 90% of the nail diseases found in toes (8). In patients on hemodialysis, the incidence of onychomycosis increases with older age (10, 11, 13), in men (10, 11, 13), and in the presence of diabetes mellitus or psoriasis (3, 5, 10–13), in renal transplant patients (10, 11), and in those infected by *human immunodeficiency virus* (HIV) (11). On the other hand, hemodialysis duration as a risk factor for onychomycosis showed divergent results in the literature (6, 10, 12, 13).

In such context, this study aimed to describe the prevalence and risk predictors for onychomycosis in patients on hemodialysis in a referral hospital in Brazil.

## 2 Methods

The study was carried out at the São Francisco de Assis University Hospital Hemodialysis Center in Bragança Paulista-SP, Brazil. The study included all patients with chronic renal insufficiency who were in regular dialysis treatment in the period from July 2022 to March 2023 and who were over 18 years old. The hemodialysis treatment was carried out in a hospital reference center at a scheduled time so that there was no overcrowding, and the same treatment protocol with similar dialysis days between patients was followed as the referenced unit is associated with severe cases only.

**Figure 1:**
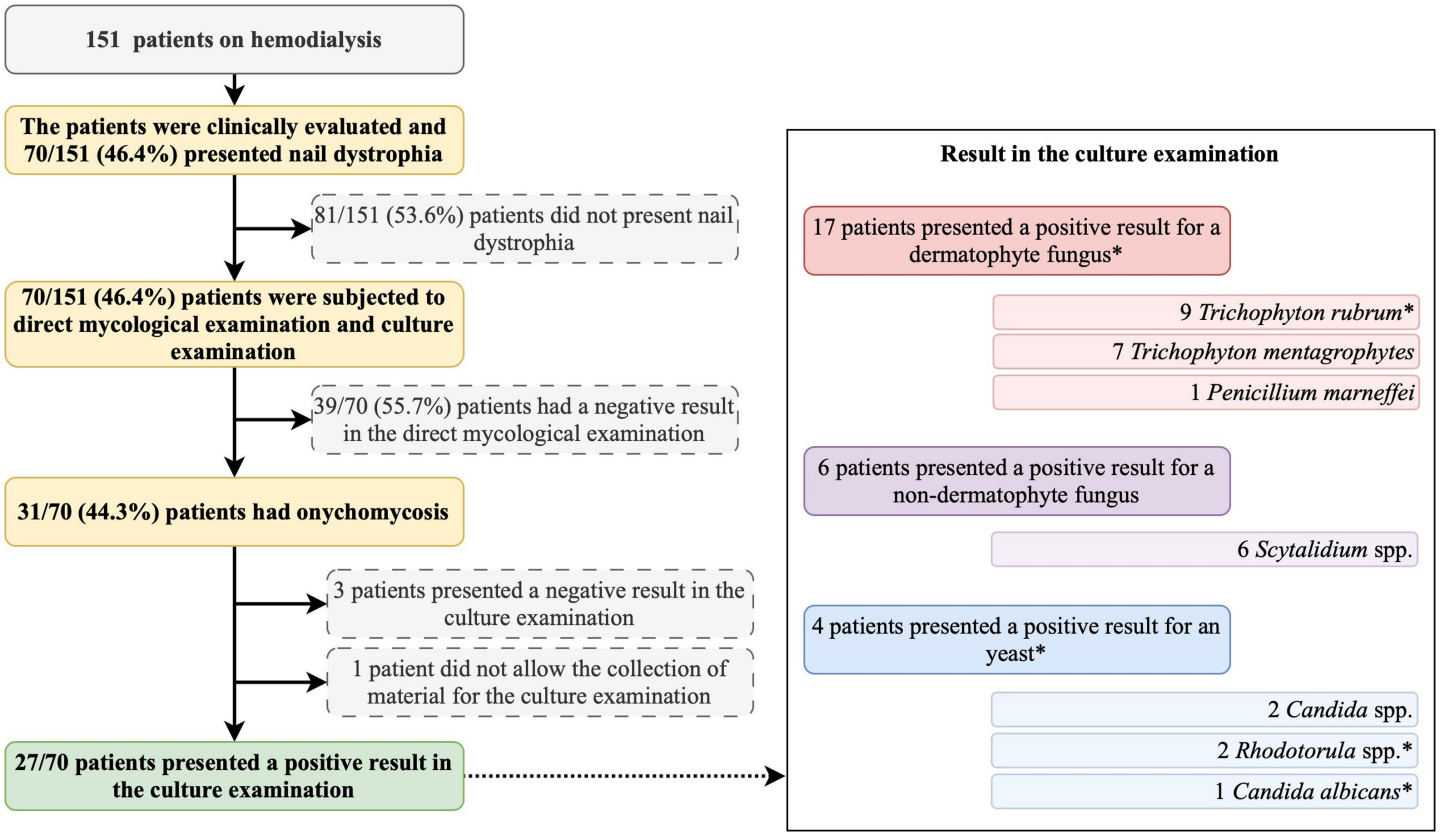
Summary of the study protocol and main findings. *, one participant presented a positive result for *Trichophyton rubrum* and *Rhodotorula* spp.

All patients were first clinically evaluated, and those presenting nail dystrophy were subjected to direct mycological examination and fungal culture (Figure 1). The material used in both examinations corresponded to subungual debris of the affected nail plate, obtained from scraping the clinically changed areas after being cleaned with 70% alcohol, using an aluminum spatula and sterile nail pliers to obtain material from the progression region and the confluence of healthy and affected tissue. The material obtained was deposited in sterile bottles and sent to the clinical analysis laboratory.

For the direct mycological examination, the material was cleared with potassium hydroxide to degrade the keratin and placed on a slide to visualize the fungal structures using an optical microscope. The analysis provided information regarding the morphology, but species identification could not be determined by the examination. Fungal culture is considered the gold standard for the diagnosis of onychomycosis since it enables the identification of the fungal species affecting the patient and shows the fungus viability. The examination uses the Sabouraud and Sabouraud cycloheximide agar culture media. The culture is established at 20–25°C, and the waiting time for growth might reach up to 4 weeks. The fungal agent identification is achieved using culture macromorphology and micromorphology analyses. The direct mycological examination and the fungal culture were carried out at the clinical analysis laboratory of the University Hospital, certified by the Brazilian Sanitation Surveillance for this type of examination. In our data, the patients on hemodialysis were considered affected by onychomycosis when the direct mycological examination presented a positive result.

The patients on hemodialysis had the following markers collected: age (years), sex (male and female), race [white, black, mixed race (*pardos*), and yellow (Asian)], schooling (illiterate, elementary school, high school, and higher education), body mass index (kg/m^2^), household location (rural or urban), presence of comorbidity, hemodialysis duration (classified in ≤1 year or > 1 year), and onychomycosis treatment before their inclusion in this study. Schooling was evaluated in our study with the aim of verifying the association of a social marker with the risk of onychomycosis due to accessibility to hemodialysis and onychomycosis treatment since our tertiary hospital is responsible for the care of patients assisted by the public and private healthcare system. For the patient on hemodialysis with a positive result in the fungal culture examination, the following markers were also evaluated: (a) clinical type, (b) nail involvement severity score, and (c) number of dystrophic nails per patient.

The classification of onychomycosis was performed according to the literature into the following groups: distal and lateral subungual, superficial, endonyx, proximal subungual, mixed, totally dystrophic, and secondary onychomycosis (14).

The onychomycosis severity was evaluated using the Onychomycosis Severity Index system. The score was obtained by multiplying the score for the area of involvement (range, 0 to 5) by the score for the proximity of disease to the matrix (range, 1 to 5). Ten points were added for the presence of a longitudinal streak or a patch (dermatophytoma) or for a patch greater than 2 mm of subungual hyperkeratosis. After measuring the scores, patients on hemodialysis were classified into the following severity groups: (a) mild onychomycosis [score of 1 through 5], (b) moderate onychomycosis [score of 6 through 15], and (c) severe onychomycosis [score of 16 through 35] (15).

The statistical analysis was carried out using the software *Statistical Package for the Social Sciences* (IBM Corp. Released 2021. IBM SPSS Statistics for Macintosh, Version 28.0. Armonk, NY: IBM Corp). In the study, the sample size was not calculated because we enrolled all patients on hemodialysis from our institution (convenience sampling). Categorical data are presented considering absolute frequency (*n*) and relative frequency (%). The association between categorical markers among those that presented positive ungual alterations and onychomycosis diagnosis and those that did not were obtained employing Pearson’s chi-square or Fisher’s exact tests. Regarding categorical data, *Odds Ratio* (OR) calculation was performed with a 95% confidence interval (95%CI), and the risk parameter was the group of participants with ungual alterations and onychomycosis-positive diagnosis. Numerical data are presented by the mean ± standard deviation and median (25–75 percentile). The association between markers with numerical distribution among those with and without ungual alterations and onychomycosis-positive diagnosis was carried out using the Mann–Whitney U-test or T-test according to the normality of data. The numerical data normality was evaluated considering three parameters: (i) analysis of the descriptive measure of central tendency, (ii) graphic method (normal Q-Q plot, Q-Q plot without tendency, and boxplot), and (iii) statistical test method (normality tests), Kolmogorov–Smirnov and Shapiro–Wilk tests. The alpha error value adopted was 0.05.

In the multivariable analysis, a binary logistic regression model with the backward stepwise method was used. In the regression model (multivariable analysis), the markers with a *p*-value of ≤0.25 in the bivariate analysis were included. The inclusion of markers with a *p*-value of ≤0.25 in the bivariate analysis was based on the Wald test from logistic regression. More traditional levels such as 0.05 can fail in identifying variables known to be important (16–18). The response variable was the onychomycosis-positive diagnosis. The multivariable analysis included eight patients’ characteristics. The logistic regression model presented the (i) B coefficient [including the SE (standard error)], which for the constant was called intercept; (ii) the Wald chi-square test and its significance; (iii) degrees of freedom (df) for the Wald chi-square test; and (iv) Exp (B) which represents the exponentiation of the B coefficient (OR) including its 95%CI. Before performing the statistical analysis, the markers were tested for multicollinearity considering cutoffs of <0.1 for tolerance and >10 for variance inflation factor. The alpha error value adopted was 0.05.

## 3 Results

This study included 151 patients on hemodialysis (Figure 1), predominantly male individuals (*n* = 79; 52.3%), white (*n* = 101; 66.9%), residing in the urban area (*n* = 118; 78.2%), and who had completed elementary school (*n* = 86; 57.0%) (Table 1). Systemic arterial hypertension (*n* = 138; 91.4%), diabetes mellitus (*n* = 56; 37.1%), and obesity (*n* = 20; 13.2%) were the main comorbidities observed (Table 1).

**Table 1:** Markers evaluated in the study on patients on hemodialysis that were examined for the presence of onychomycosis.

Patients’ characteristics	n/N (%) or mean ± standard deviation; median (p25 to p75)
Sex	
Female	72/151 (47.7%)
Male	79/151 (52.3%)
Race	
White	101/151 (66.9%)
Black	20/151 (13.2%)
*Pardo* (mixed race)	24/151 (15.9%)
Asian	6/151 (4.0%)
Age (years)	57.64 ± 15.36; 60 (46 to 70)
Body mass index (Kg/m^2^)	24.63 ± 5.21; 24 (22 to 28)
Schooling	
Illiterate	13/151 (8.6%)
Elementary school	86/151 (57.0%)
High school	43/151 (28.5%)
Higher education	9/151 (6.0%)
Household location	
Urban	118/151 (78.2%)
Rural	33/151 (21.8%)
Comorbidity	
Systemic arterial hypertension	138/151 (91.4%)
Diabetes mellitus	56/151 (37.1%)
Obesity	20/151 (13.2%)
Cardiopathy	15/151 (9.9%)
Thyroidopathy	8/151 (5.3%)
Others	20/151 (13.2%)
Hemodialysis duration	
≤1 year	64/151 (42.4%)
>1 year	87/151 (57.6%)
Individuals with ungual alteration	
Yes	70/151 (46.4%)
No	81/151 (53.6%)
Previous medication treatment	
Yes	15/151 (9.9%)
No	136/151 (90.1%)
Mycological exam result	
Positive	31/70 (46.3%)
Negative	36/70 (53.7%)
Culture result*	
*Trichophyton rubrum* (dermatophyte fungus)	9/31 (29.0%)
*Trichophyton mentagrophytes* (dermatophyte fungus)	7/31 (22.6%)
*Scytalidium* spp. (non-dermatophyte fungus)	6/31 (19.4%)
*Candida* spp. (yeast)	2/31 (6.5%)
*Rhodotorula* spp. (yeast)	2/31 (6.5%)
*Candida albicans* (yeast)	1/31 (3.2%)
*Penicillium marneffei* (dermatophyte fungus)	1/31 (3.2%)
Negative	3/31 (9.7%)
Refused (inconclusive)	1/31 (3.2%)

*One participant presented a positive result for *T. rubrum* and *Rhodotorula* spp. *N*, number of patients; p25, percentile 25%; p75, percentile 75%; %, percentage; ≤, less than or equal to; >, greater than.

Hemodialysis was associated, respectively, with secondary chronic renal disease and systemic arterial hypertension (*n* = 58; 37.9%), systemic arterial hypertension and diabetes mellitus (*n* = 33; 21.9%), diabetes mellitus (*n* = 19; 12.6%), glomerulopathy (*n* = 12; 7.9%), polycystic renal disease (*n* = 9; 6.0%), nephritis (*n* = 8; 5.3%), focal segmental glomerulosclerosis (*n* = 7; 4.6%), obstructive nephropathy (*n* = 4; 2.6%), and vasculitis (*n* = 1; 0.7%).

Among the study participants, 70 (46.4%) showed ungual alterations (Figure 1), and only 15 (9.9%) of those evaluated had used some medication to treat onychomycosis, and out of them, only one reported systemic use of the medication (Table 1). In the mycological exam, 31 (44.3%) out of the 70 examined participants had a positive result (Figure 1). According to the microbiological culture result, 8 (25.8%) participants presented *T. rubrum*, 7 (22.6%) presented *T. mentagrophytes*, 6 (19.4%) presented *Scytalidium* spp., 2 (6.5%) presented *Candida* spp., 1 (3.2%) presented *Rhodotorula* spp., 1 (3.2%) presented *Candida albicans*, 1 (3.2%) presented *Penicillium marneffei*, and 1 (3.2%) presented *T. rubrum* and *Rhodotorula* spp. colony growth (Figure 1). Three participants presented negative results in the culture examination, and one did not allow the collection of material for the examination (Figure 1).

For the patients on hemodialysis and with a positive result in the fungal culture examination, the nail involvement severity score for the majority of them was severe [23 out of 27 (85.2%)], and only 1 out of 27 (3.7%) and 3 out of 27 (1.1%) patients presented moderate and mild scores, respectively. In addition, for those patients on hemodialysis, distal subungual onychomycosis occurred in 12 out of 27 (44.4%) patients, mixed pattern in 14 out of 27 (51.9%) patients, and, white superficial onychomycosis in only 1 out of 27 (3.7%) patients (Table 2).

**Table 2:** Clinical type, nail involvement severity score, and number of dystrophic nails per patient on hemodialysis with a positive result in the fungal culture examination.

Culture result	Microorganisms’ classification	Clinical type (hallux)	Nail involvement severity score*	Dystrophic nails (N)
*Candida albicans*	Yeast	Distal subungual	Severe	2
*Candida* spp.	Yeast	Mixed pattern	Severe	6
*Candida* spp.	Yeast	Mixed pattern	Severe	1
*Penicillium marneffei*	Non-dermatophyte fungus	Distal subungual	Severe	4
*Rhodotorula* spp.	Yeast	White superficial	Mild	8
*Scytalidium* spp.	Non-dermatophyte fungus	Mixed pattern	Severe	10
*Scytalidium* spp.	Non-dermatophyte fungus	Distal subungual	Severe	10
*Scytalidium* spp.	Non-dermatophyte fungus	Mixed pattern	Severe	10
*Scytalidium* spp.	Non-dermatophyte fungus	Distal subungual	Severe	1
*Scytalidium* spp.	Non-dermatophyte fungus	Mixed pattern	Moderate	2
*Scytalidium* spp.	Non-dermatophyte fungus	Distal subungual	Severe	9
*Trichophyton mentagrophytes*	Dermatophyte fungus	Mixed pattern	Mild	3
*Trichophyton mentagrophytes*	Dermatophyte fungus	Distal subungual	Severe	3
*Trichophyton mentagrophytes*	Dermatophyte fungus	Distal subungual	Severe	6
*Trichophyton mentagrophytes*	Dermatophyte fungus	Distal subungual	Severe	4
*Trichophyton mentagrophytes*	Dermatophyte fungus	Mixed pattern	Severe	2
*Trichophyton mentagrophytes*	Dermatophyte fungus	Mixed pattern	Severe	10
*Trichophyton mentagrophytes*	Dermatophyte fungus	Distal subungual	Mild	2
*Trichophyton rubrum*	Dermatophyte fungus	Distal subungual	Severe	4
*Trichophyton rubrum*	Dermatophyte fungus	Mixed pattern	Severe	9
*Trichophyton rubrum*	Dermatophyte fungus	Mixed pattern	Severe	4
*Trichophyton rubrum*	Dermatophyte fungus	Mixed pattern	Severe	10
*Trichophyton rubrum*	Dermatophyte fungus	Distal subungual	Severe	5
*Trichophyton rubrum*	Dermatophyte fungus	Distal subungual	Severe	2
*Trichophyton rubrum*	Dermatophyte fungus	Mixed pattern	Severe	10
*Trichophyton rubrum*	Dermatophyte fungus	Mixed pattern	Severe	9
*Trichophyton rubrum* + *Rhodotorula* spp.	Dermatophyte fungus + Yeast	Mixed pattern	Severe	6

N, number of patients. *The onychomycosis severity was evaluated using the Onychomycosis Severity Index system. The score was obtained by multiplying the score for the area of involvement (range, 0 to 5) by the score for the proximity of disease to the matrix (range, 1 to 5). Ten points were added for the presence of a longitudinal streak or a patch (dermatophytoma) or for greater than 2 mm of subungual hyperkeratosis. Mild onychomycosis corresponds to a score of 1 through 5; moderate, 6 through 15; and severe, 16 through 35 (15).

**Figure 2:**
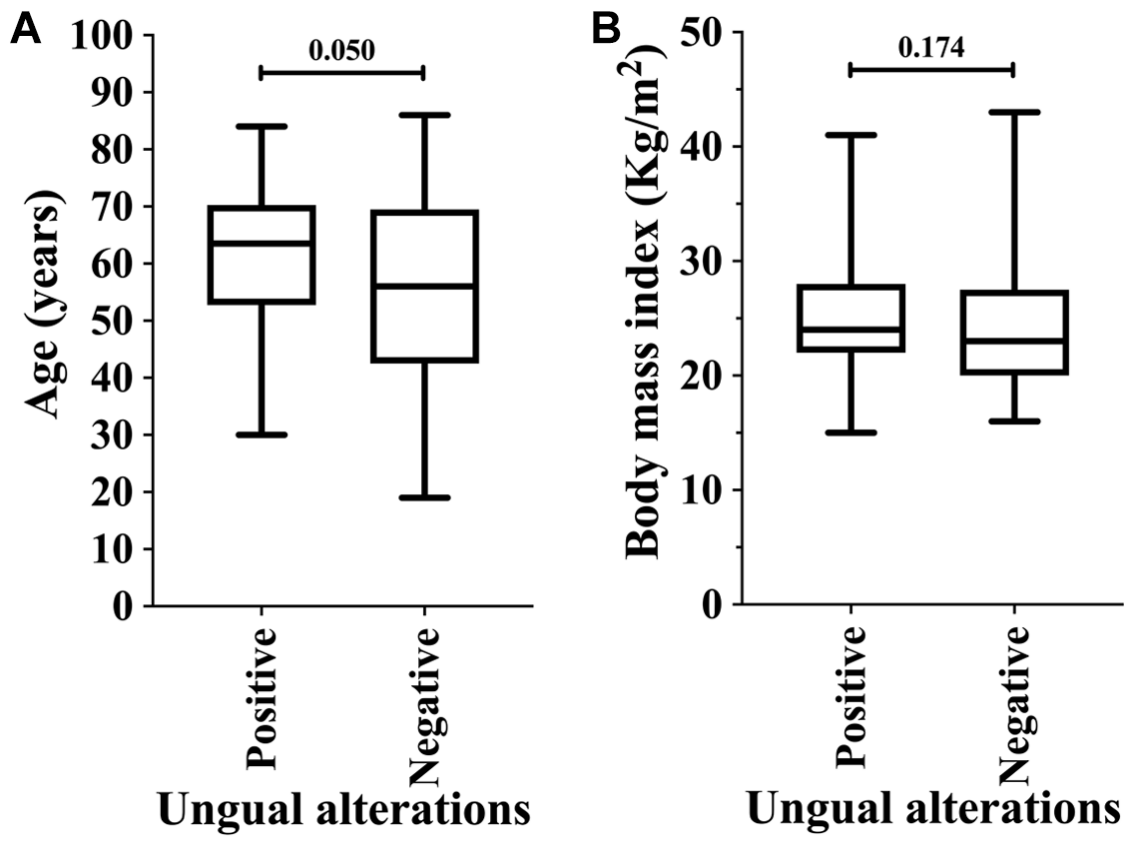
Association of ungual alterations in patients on hemodialysis in relation to age (years) **(A)** and body mass index **(B)**. An alpha error of 0.05 was adopted in both analyses. The statistical analysis was carried out using the Mann–Whitney U-test or T-test according to the normality of data.

In our data, the male sex was associated with the higher prevalence of ungual alterations [43/70 (61.4%) vs. 36/81 (44.4%); OR = 1.991 (95%CI = 1.038 to 3.817)]; moreover, other markers such as race, schooling, comorbidities, household location, and hemodialysis period were not associated as risk factors (Table 3). Body mass index did not show a statistically significant difference between the groups of participants regarding the ungual alterations (*p*-value = 0.174; 24.0 Kg/m^2^ vs. 23.0 Kg/m^2^) (Figure 2B). Patients on hemodialysis with ungual alterations were older (*p*-value = 0.050; 62.5 years old vs. 56.0 years old) (Figure 2A).

**Table 3:** Association between markers evaluated in the study and risk of ungual alterations in patients on hemodialysis of a tertiary hospital.

Marker	Group	Positive	Negative	Total	*P*-value	OR	95%CI
Sex	Male	43 (61.4%)	36 (44.4%)	79 (52.3%)	**0.050**^**a**^	**1.991**	**1.038 to 3.817**
	Female	27 (38.6%)	45 (55.6%)	72 (47.7%)		1	Reference
Race	White	48 (68.6%)	53 (65.4%)	101 (66.9%)	0.543^b^	1.153	0.583 to 2.278
	*Pardo* (mixed race)	12 (17.1%)	12 (14.8%)	24 (15.9%)		1.190	0.497 to 2.848
	Black	9 (12.9%)	11 (13.6%)	20 (13.2%)		0.939	0.365 to 2.417
	Asian	1 (1.4%)	5 (6.2%)	6 (4.0%)		0.222	0.005 to 2.056
Schooling	Illiterate	7 (10.0%)	6 (7.4%)	13 (8.6%)	0.270^b^	1.389	0.444 to 4.346
	Elementary school	44 (62.9%)	42 (51.9%)	86 (57.0%)		1.571	0.819 to 3.016
	High school	17 (24.3%)	26 (32.1%)	43 (28.5%)		0.679	0.331 to 1.392
	Higher education	2 (2.9%)	7 (8.6%)	9 (6.0%)		0.313	0.031 to 1.721
Comorbidities							
Systemic arterial hypertension	Yes	66 (94.3%)	72 (88.9%)	138 (91.4%)	0.263^b^	2.053	0.541 to 9.563
	No	4 (5.7%)	9 (11.1%)	13 (8.6%)		1	Reference
Diabetes mellitus	Yes	28 (40.0%)	28 (34.6%)	56 (37.1%)	0.504^a^	1.262	0.651 to 2.446
	No	42 (60.0%)	53 (65.4%)	95 (62.9%)		1	Reference
Obesity	Yes	10 (14.3%)	10 (12.3%)	20 (13.2%)	0.812^a^	1.183	0.462 to 3.034
	No	60 (85.7%)	71 (87.7%)	131 (86.8%)		1	Reference
Cardiopathy	Yes	7 (10.0%)	8 (9.9%)	15 (9.9%)	1.000^a^	1.014	0.348 to 2.952
	No	63 (90.0%)	73 (90.1%)	136 (90.1%)		1	Reference
Thyroidopathy	Yes	6 (8.6%)	2 (2.5%)	8 (5.3%)	0.145^b^	3.673	0.629 to 38.41
	No	64 (91.4%)	79 (97.5%)	143 (94.7%)		1	Reference
Other comorbidities	Yes	11 (15.7%)	9 (11.1%)	20 (13.2%)	0.474^a^	1.492	0.579 to 3.840
	No	59 (84.3%)	72 (88.9%)	131 (86.8%)		1	Reference
Household location	Urban	57 (81.4%)	61 (75.3%)	118 (78.1%)	0.432^a^	1.438	0.655 to 3.155
	Rural	13 (18.6%)	20 (24.7%)	33 (21.9%)		1	Reference
Hemodialysis period	≤1 year	26 (37.1%)	38 (46.9%)	64 (42.4%)	0.251^a^	0.669	0.348 to 1.284
	>1 year	44 (62.9%)	43 (53.1%)	87 (57.6%)		1	Reference

OR, Odds Ratio; 95%CI, 95% confidence interval; ≤, less than or equal to; >, greater than. ^a^Pearson’s chi-square test. ^b^Fisher’s Exact test. Significant data are presented in bold. An alpha error of 0.05 was adopted in all analyses carried out.

**Figure 3:**
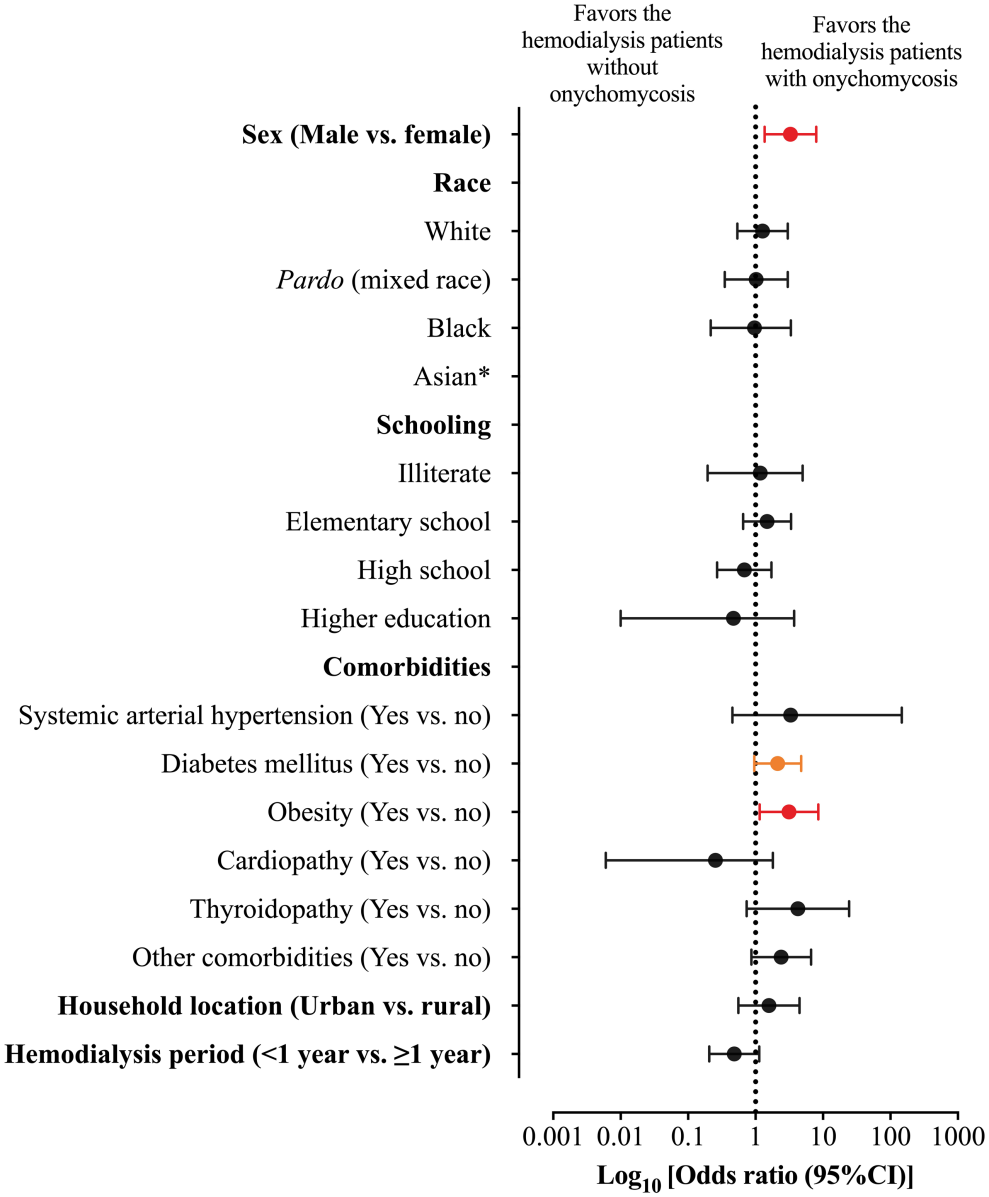
Odds ratio (OR) and 95% confidence interval (95%CI) for the association between markers evaluated in the study and risk of onychomycosis-positive diagnosis in patients on hemodialysis of a tertiary hospital. *, the OR was not imputed because no patient was classified as Asiatic in the group of onychomycosis-positive diagnosis. The red color represents the markers (gender and obesity) with a statistical significance difference between groups and with a significant 95%CI which did not include one. The orange color represents the marker (diabetes mellitus) with a statistical significance difference between groups and with a significant 95%CI which included one.

**Figure 4:**
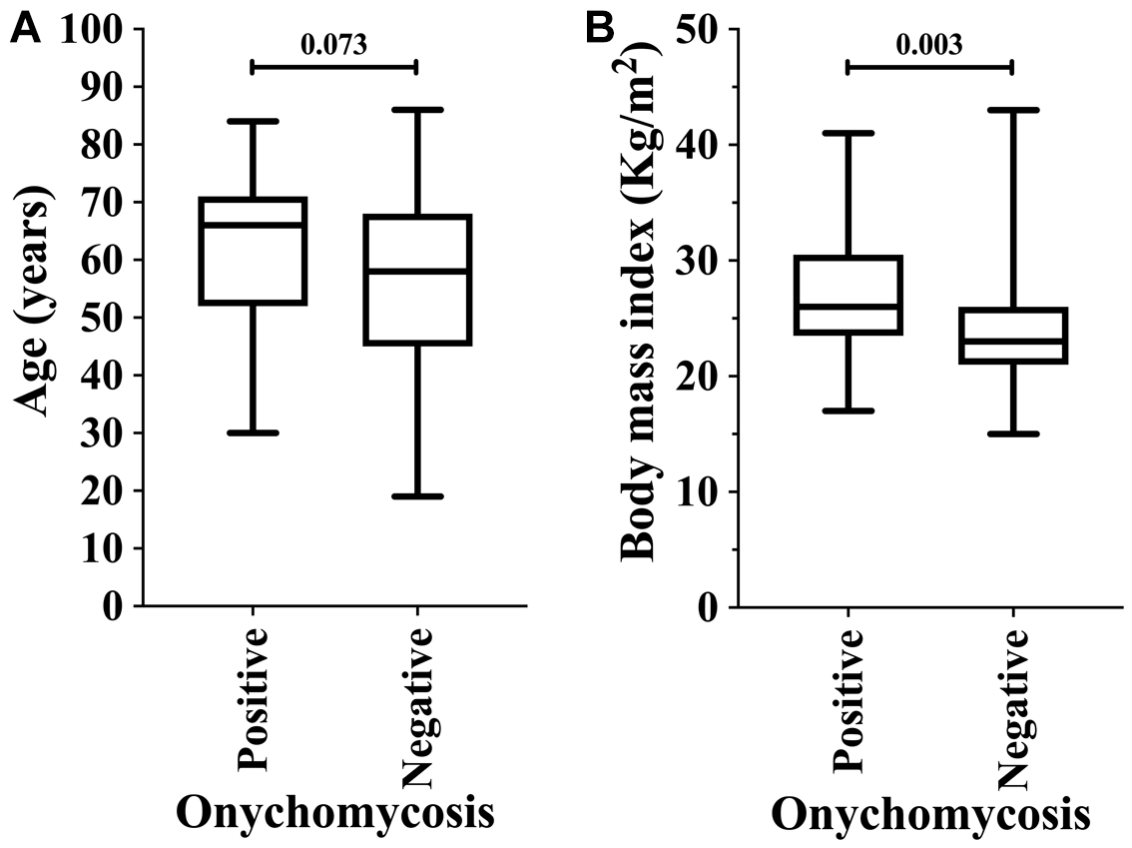
Association of onychomycosis-positive diagnosis in patients on hemodialysis in relation to age (years) **(A)** and body mass index **(B)**. An alpha error of 0.05 was adopted in both analyses. The statistical analysis was carried out using the Mann–Whitney U-test or T-test according to the normality of data.

Among the markers evaluated in this study, those associated with higher risk of onychomycosis were male sex [23/31 (74.2%) vs. 56/120 (46.7%); OR = 3.286 (95%CI = 1.362 to 7.928)] and obesity [8/31 (25.8%) vs. 12/120 (10.0%); OR = 3.130 (95%CI = 1.150 to 8.521)]. At the same time, patients with diabetes mellitus were more susceptible to the onychomycosis attack [*p*-value = 0.049; 16/31 (51.6%) vs. 40/120 (33.3%); however, OR was 2.133 (95%CI = 0.959 to 4.648)] (Table 4; Figure 3). Participants with onychomycosis-positive diagnosis presented higher body mass index (*p*-value = 0.003; 26.0 Kg/m^2^ vs. 23.0 Kg/m^2^) (Figure 4B). Age did not show a statistically significant difference between the groups of participants regarding the onychomycosis diagnosis (*p*-value = 0.073; 66 years old vs. 58 years old) (Figure 4A).

**Table 4:** Association between markers evaluated in the study and risk of onychomycosis-positive diagnosis in patients on hemodialysis of a tertiary hospital.

Marker	Group	Positive	Negative	Total	*P*-value	OR	95%CI
Sex	Male	23 (74.2%)	56 (46.7%)	79 (52.3%)	**0.008**^**a**^	**3.286**	**1.362 to 7.928**
	Female	8 (25.8%)	64 (53.3%)	72 (47.7%)		1	Reference
Race	White	22 (71.0%)	79 (65.8%)	101 (66.9%)	0.825^b^	1.269	0.536 to 3.005
	*Pardo* (mixed race)	5 (16.1%)	19 (15.8%)	24 (15.9%)		1.022	0.349 to 2.996
	Black	4 (12.9%)	16 (13.3%)	20 (13.2%)		0.963	0.217 to 3.330
	Asian	0 (0.0%)	6 (5.0%)	6 (4.0%)		NA	NA
Schooling	Illiterate	3 (9.7%)	10 (8.3%)	13 (8.6%)	0.795^b^	1.177	0.195 to 4.994
	Elementary school	20 (64.5%)	66 (55.0%)	86 (57.0%)		1.488	0.656 to 3.374
	High school	7 (22.6%)	36 (30.0%)	43 (28.5%)		0.681	0.269 to 1.721
	Higher education	1 (3.2%)	8 (6.7%)	9 (6.0%)		0.469	0.010 to 3.732
Comorbidities							
Systemic arterial hypertension	Yes	30 (96.8%)	108 (90.0%)	138 (91.4%)	0.306^b^	3.314	0.455 to 147.2
	No	1 (3.2%)	12 (10.0%)	13 (8.6%)		1	Reference
Diabetes mellitus	Yes	16 (51.6%)	40 (33.3%)	56 (37.1%)	**0.049**^**a**^	2.133	0.959 to 4.748
	No	15 (48.4%)	80 (66.7%)	95 (62.9%)		1	Reference
Obesity	Yes	8 (25.8%)	12 (10.0%)	20 (13.2%)	**0.027**^**a**^	**3.130**	**1.150 to 8.521**
	No	23 (74.2%)	108 (90.0%)	131 (86.8%)		1	Reference
Cardiopathy	Yes	1 (3.2%)	14 (11.7%)	15 (9.9%)	0.141^b^	0.254	0.006 to 1.803
	No	30 (96.8%)	106 (88.3%)	136 (90.1%)		1	Reference
Thyroidopathy	Yes	4 (12.9%)	4 (3.3%)	8 (5.3%)	0.056^b^	4.241	0.741 to 24.32
	No	27 (87.1%)	116 (96.7%)	143 (94.7%)		1	Reference
Other comorbidities	Yes	7 (22.6%)	13 (10.8%)	20 (13.2%)	0.082^**a**^	2.401	0.866 to 6.657
	No	24 (77.4%)	107 (89.2%)	131 (86.8%)		1	Reference
Household location	Urban	26 (83.9%)	92 (76.6%)	118 (78.1%)	0.387^**a**^	1.583	0.556 to 4.506
	Rural	5 (16.1%)	28 (23.3%)	33 (21.9%)		1	Reference
Hemodialysis period	≤1 year	9 (29.0%)	55 (45.8%)	64 (42.4%)	0.068^**a**^	0.484	0.206 to 1.136
	>1 year	22 (71.0%)	65 (54.2%)	87 (57.6%)		1	Reference

NA, not applicable; OR, Odds Ratio; 95%CI, 95% confidence interval; ≤, less than or equal to; >, greater than. ^a^Pearson’s chi-square test. ^b^Fisher’s exact test. Significant data are presented in bold. An alpha error of 0.05 was adopted in all analyses carried out.

The multivariable model using the logistic regression (backward model) was able to predict (81.5%) the onychomycosis-positive diagnosis (chi-square = 27.73; degrees of freedom = 5; *p*-value <0.001; R^2^ Nagelkerke = 0.263) (Table 5). The older age [OR = 1.036; 95% CI = 1.004 to 1.069], male sex [OR = 5.746; 95%CI = 2.057 to 16.046], and presence of obesity [OR = 4.800; 95% CI = 1.435 to 16.055] were positive and significant in predicting the onychomycosis-positive diagnosis (Table 5).

**Table 5:** Multivariable analysis to determine the association between markers evaluated in the study and risk of onychomycosis-positive diagnosis in patients on hemodialysis of a tertiary hospital.^a,*^

Markers	B	SE	Wald	df	*P*-value	*Odds ratio*	95%CI
Age (years)	0.035	0.016	4.854	1	**0.028**	**1.036**	**1.004 to 1.069**
Sex (male)	1.748	0.524	11.135	1	**0.001**	**5.746**	**2.057 to 16.046**
Comorbidities (yes)							
Obesity	1.569	0.616	6.483	1	**0.011**	**4.800**	**1.435 to 16.055**
Cardiopathy	−1.941	1.118	3.017	1	0.082	0.144	0.016 to 1.283
Thyroidopathy	1.633	0.887	3.391	1	0.066	5.121	0.900 to 29.132
Constant	−4.757	1.112	18.314	1	<0.001	0.009	

SE, standard error; 95%CI, 95% confidence interval; df, degrees of freedom. In the multivariable analysis, it was used the binary logistic regression model with the backward stepwise method. Significant data are presented in bold. An alpha error of 0.05 was adopted in all analyses carried out. ^a^Markers included in step one: age, gender, comorbidities (obesity, cardiopathy, thyroidopathy, and other comorbidities), and hemodialysis period. ^*^It was included in the regression model (multivariable analysis) the markers with a *p*-value of ≤0.25 in the bivariate analysis (Table 4).

**Figure 5:**
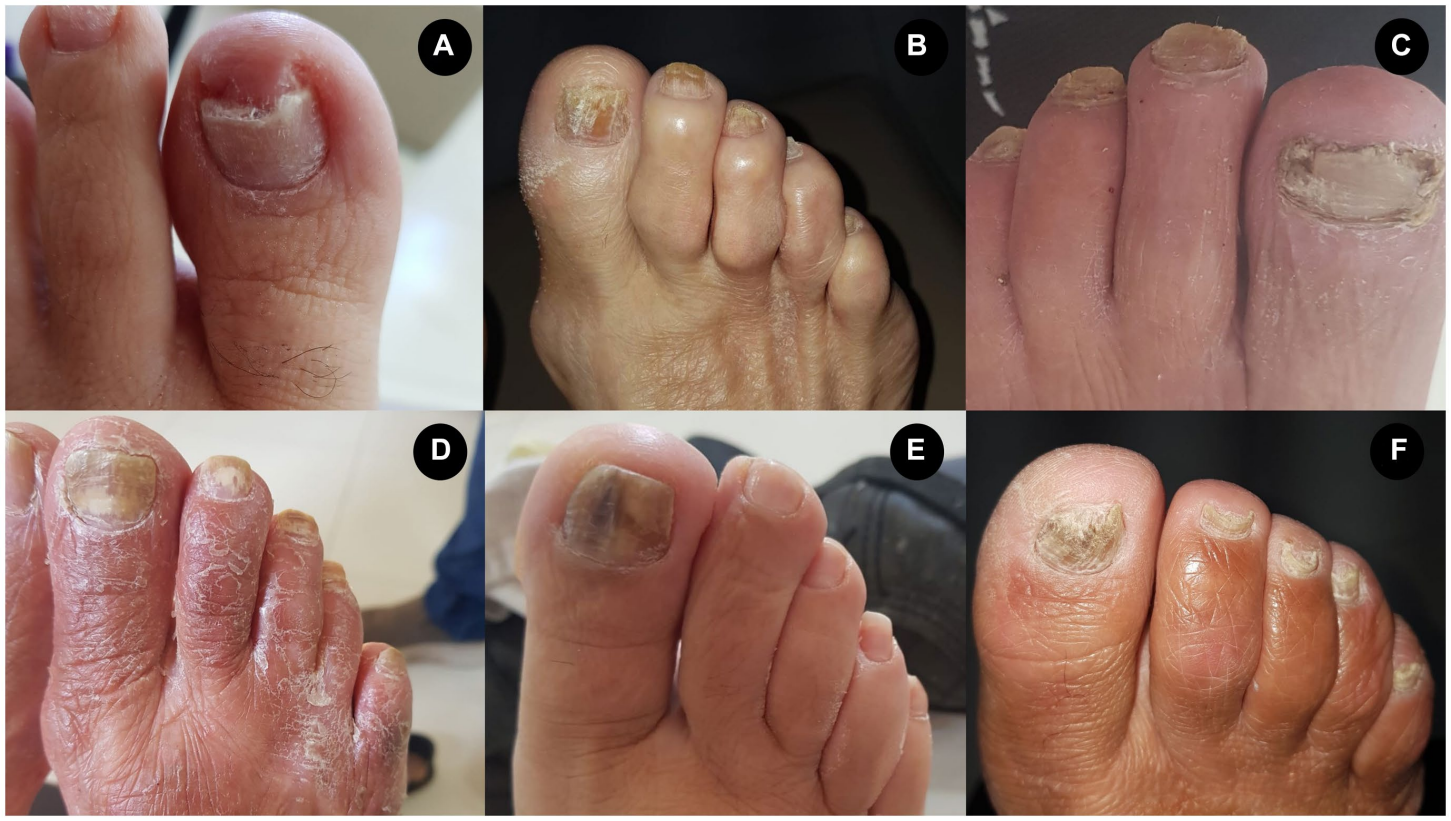
Presentation of different phenotypes associated with onychomycosis in patients on hemodialysis. **(A)** *Candida albicans* (yeast). **(B)** *Trichophyton rubrum* (dermatophyte fungus). **(C)** *Scytalidium* spp. (non-dermatophyte fungus). **(D)** *T. rubrum* (dermatophyte fungus) and *Rhodotorula* spp. (yeast). **(E)** *Candida* spp. (yeast). **(F)** *Trichophyton mentagrophytes* (dermatophyte fungus).

**Figure 6:**
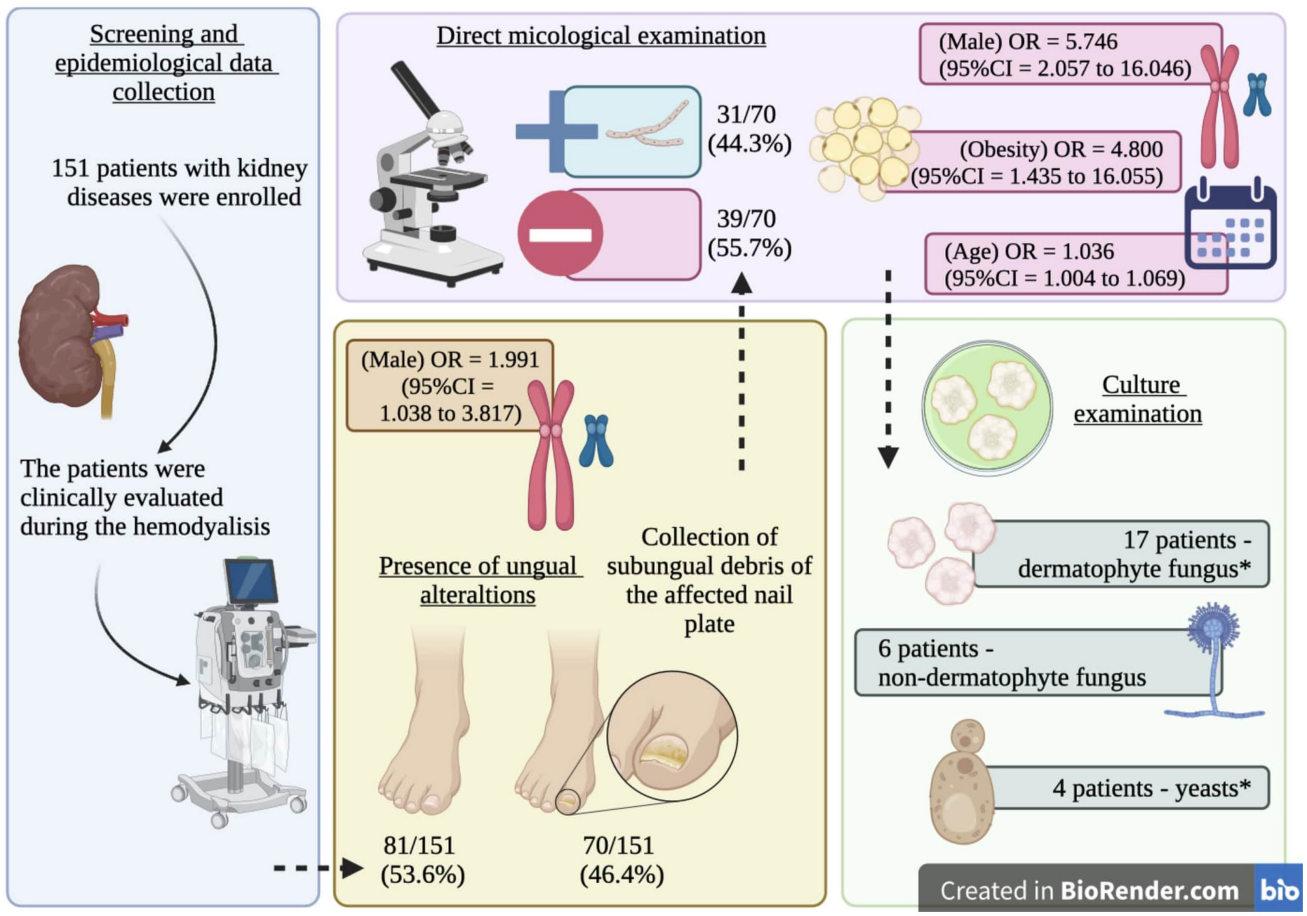
Graph summary. Scheme of the study protocol and main findings including the *Odds ratio* (OR) and 95% confidence intervals for the risk factors of ungual alterations (gender) and onychomycosis (age, gender, and obesity) in patients on hemodialysis. *, one participant presented a positive result for *Trichophyton rubrum* and *Rhodotorula* spp.

Some clinical phenotypes of onychomycosis in the patients on hemodialysis included in the study are presented below (Figure 5); in addition, an overview of our study protocol and main findings are presented in Figure 6.

## 4 Discussion

Patients with chronic renal disease on hemodialysis presented a high risk of developing onychomycosis when compared to the general population. Most of the patients evaluated in this study were male individuals, and the comorbidities frequently associated with these patients on hemodialysis were systemic arterial hypertension, diabetes mellitus, obesity, cardiopathy, and tyroidopathy, while male sex and the presence of obesity and diabetes mellitus were risk factors for onychomycosis. Among the 151 patients evaluated, 70 out of 151 (46.4%) showed ungual alterations and, among them, 27 out of 70 (38.5%) obtained positive direct mycological examination and culture. When investigating the positive cultures, a predominance of dermatophyte fungi was observed, followed by non-dermatophyte fungi and yeasts, and the etiological agents were, respectively, *T. rubrum*, *T. mentagrophytes*, *P. marneffei*, *Scytadilium* spp., *Candida* spp., *Rhodotorula* spp., and *C. albicans*.

In the literature, a review was carried out aiming at determining the prevalence of onychomycosis in high-risk patients, that is, children, older individuals, patients with diabetes mellitus, individuals with psoriasis, HIV+, individuals with renal transplant, and those on hemodialysis. Out of the patients evaluated, 109 patients were on hemodialysis and the most common onychomycosis presentation was the distal-lateral subungual, while the most prevalent etiological agents were filamentous and dermatophyte fungi, while yeast prevailed in the elderly, patients with diabetes mellitus, and individuals with psoriasis. Older patients with diabetes mellitus, psoriasis, HIV+, on hemodialysis, and with renal transplants showed high onychomycosis prevalence. Reports were found describing that the factors that might influence these results are damaged immunity, reduced peripheral circulation, and ungual dystrophy (11).

In our data, we identified *P. marneffei* in one patient on hemodialysis, which is an opportunistic fungal species mainly for immunocompromised patients, such as HIV+ patients, and can cause the penicilliosis disease (19, 20). This disease was first identified in Thailand and Southeast Asia, and it affects the skin, mucosa, lungs, pleura, lymph nodes, central nervous system, and bone marrow, and the risk increases with exposure to soil (21, 22). This disseminated fungal infection causes high morbidity and mortality (19). This fungus is uncommon in onychomycosis and immunocompetent patients in the general population, and it was first described by Gupta (23) in a 60-year-old man who had onychomycosis diagnostic in the fingernail, and the only risk factor was gardening. To the best of our knowledge, this infection has never been described in Brazil (24).

The incidence of superficial fungal infections is related to genetic susceptibility, family background (25, 26), and environmental factors such as moist, wearing shoes, excessive sweat, and cross-contamination by other contaminated individuals and contaminated objects and surfaces (27). These factors associated with low immunity predispose the individual to onychomycosis development. Chronic renal disease tends to occur simultaneously with high uremia, which increases oxygen-reactive species, leading to oxidative stress (28). In addition, patients on hemodialysis show a higher pro-inflammatory response occurring with increased expression of polymorphonuclear neutrophils of the TRL2 (*Toll-like Receptor 2*) and TRL4 (*Toll-like Receptor 4*) receptors of monocytes. They also decrease the number and function of dendritic cells reducing the innate immunologic system expression, in addition to harming the adaptive immunologic system with a reduction in the number and function of T CD4+ (cluster of differentiation 4) and CD8+ (cluster of differentiation 8) cells as well as B cells. Thus, renal function loss reduces the effectiveness of the immunologic system and creates a pro-inflammatory environment with important consequences such as endothelial dysfunction, systemic inflammation, and predisposition to infections, including fungal infections (29).

Male sex is considered a risk factor for the development of onychomycosis in patients on hemodialysis (10, 11, 13) and in the general population (30–33); men present approximately 2.99 more chances of acquiring fungal infection when compared to women (34). However, despite the several reports in the literature addressing its predominance in male patients, there is no consensus about the explanation for this fact. It might be associated with immunity reduction and genetic and environmental factors, as previously mentioned. Moreover, women seek medical assistance to solve skin problems more often than men, which might modulate the onychomycosis prevalence according to gender (35). There are reports about *Trichophyton* spp. and *Microsporum* spp. presenting cytosolic proteins specifically and with great affinity to progesterone, which in turn inhibits the growth of dermatophyte fungi in a dose-dependent manner since anthropophilic species respond better to steroids than geophilic species (36).

Diabetes mellitus is considered one of the main risk factors for onychomycosis among patients on hemodialysis (3, 10, 12, 13) and among the general population (7, 37–40). As described by Lamb et al. (13), patients with diabetes mellitus show 88% more risk of acquiring onychomycosis than those without that disease, and these data confirm the findings reported by Gupta et al. (34), who reported that the risk of a diabetes mellitus patient acquiring onychomycosis is 2.77 higher than that of the general population. This value is even higher in male patients with diabetes mellitus (~2.99 times). In this study, the onychomycosis severity was also related to the time of duration of the diabetes mellitus (34). Gupta et al. (34) reported other factors related to onychomycosis, the presence of this disease in the family background is among them along with the use of immunosuppressive therapy, reduction or absence of the foot dorsal artery and the posterior tibial artery pulse, and capillary filling and ankle-brachial pressure index reduction (34). The neuropathy associated with diabetes mellitus reduces skin sensitivity; thus, small traumas might remain unnoticed and when not cured, they might remain a point of entry for infections. This is associated with immunity reduction and microangiopathy results among other complications in the development of onychomycosis (41). Moreover, a neuroischemic foot increases the prevalence of onychomycosis (42).

Obesity is a risk factor for onychomycosis (37, 38, 43, 44) representing one of the most predominant factors for this disease (45); moreover, the increases in body mass index and the persistent overweight has increased the incidence of onychomycosis (46). According to Gulcan et al. (47), the association of excess fat tissue accumulation, which alters the local microvasculature and causes more sweating, also creates favorable conditions for fungal infections (47). Moreover, some authors reported an association between the presence of onychomycosis in adult patients with the coexistence of risk factors such as obesity and diabetes mellitus (43, 45).

Hemodialysis duration as a risk factor for onychomycosis is not well established in the literature, and our study revealed that it is not a risk factor, which agrees with some previous studies (10, 13); however, other studies do not agree with these data (12).

According to the etiological agents found in this study and other reports in the literary review, dermatophyte fungi were the most prevalent, followed by non-dermatophyte fungi and yeasts (8, 9). Lamb et al. (13) reported dermatophyte fungi (69.2%) as the main cause, followed by non-dermatophyte fungi (15.4%) and yeast (15.4%), and the main fungi found in the cultures were *T. interdigitale* (*n* = 12)*, Candida* spp. (*n* = 6), and *T. rubrum* (*n* = 4). Filho et al. (10) reported dermatophyte fungi (80.3%) as the main cause, while yeast (18%) appeared as the second main cause, and the minority of cases was associated with non-dermatophyte fungi (1.6%). The main etiological agents found in the culture were *T. rubrum* (39.1%)*, C. parapsilosis* (30.4%), and *T. mentagrophytes* (21.7%) in patients on hemodialysis. Kuvandik et al. (12) also found dermatophyte fungi (86.7%) as the main cause associated with onychomycosis, followed by non-dermatophyte fungi (13.3%). However, yeast was not identified, and the positive cultures presented mainly *T. rubrum* (9.2%) and *T. mentagrophytes* (1.8%) species (12). The positive culture result in this study included dermatophyte fungi *T. rubrum* (29%), *T. mentagrophytes* (22.6%), and *P. marneffei* (3.2%), the non-dermatophyte fungi *Scytalidium* spp. (19.4%), and the yeasts *Candida* spp. (6.5%), *Rhodotorula* spp. (6.5%), and *C. albicans* (3.2%), and these values agree with other studies that reported fungi *T. rubrum* and *T. mentagrophytes* as the main dermatophyte fungi and *Candida* spp. as the main cause among yeasts (8, 10, 12).

This study has some limitations, for example, the low number of participants, the inclusion of only one research center, the identification of the agents causing onychomycosis restricted to the direct microbiological and culture exams, and patient data collected using interviews and medical records. In our study, it was not possible to assess the presence of onychomycosis before hemodialysis. The population that was included belonged to the low-income class, and all participants were evaluated, free of charge, upon inclusion in the study. After inclusion, all patients with nail alterations, mainly with onychomycosis, are being followed up and, with the results of the study, we intend to implement routine follow-up of these patients by a dermatologist. In addition, the literature presents few studies on this theme, which hampers the comparative analysis of the epidemiological profile associated with onychomycosis in patients on hemodialysis with other studies.

## 5 Conclusion

In our study, onychomycosis in patients on hemodialysis was associated with a great variety of microorganisms, mainly *Trichophyton* species, and the main risk factors associated with this infection are male sex, older age, and the presence of obesity. The main fungi associated with onychomycosis in patients on hemodialysis were the dermatophyte fungi (*T. rubrum* and *T. mentagrophytes*), followed by non-dermatophyte fungi (*Scytalidium* spp.), and yeast. The nail involvement severity score for the majority of patients was severe, and distal subungual onychomycosis and mixed pattern onychomycosis were the most prevalent clinical types. Since this fungal infection can provoke drastic consequences such as amputation in patients on hemodialysis and a social constraint factor, it is important to evaluate the treatment of this condition to improve their quality of life.

## Data availability statement

The raw data supporting the conclusions of this article will be made available by the authors, without undue reservation.

## Ethics statement

The studies involving humans were approved by Comitê de Ética da Universidade São Francisco. The studies were conducted in accordance with the local legislation and institutional requirements. The participants provided their written informed consent to participate in this study.

## Author contributions

JB: Conceptualization, Formal analysis, Funding acquisition, Investigation, Methodology, Project administration, Resources, Supervision, Validation, Visualization, Writing – original draft, Writing – review & editing. MC: Conceptualization, Investigation, Methodology, Resources, Validation, Visualization, Writing – original draft, Writing – review & editing. FM: Conceptualization, Data curation, Formal analysis, Funding acquisition, Investigation, Methodology, Project administration, Resources, Supervision, Validation, Visualization, Writing – original draft, Writing – review & editing.
